# Multidisciplinary amyloidosis care in the era of personalized medicine

**DOI:** 10.3389/fneur.2022.935936

**Published:** 2022-10-13

**Authors:** Naresh Bumma, Rami Kahwash, Samir V. Parikh, Michael Isfort, Miriam Freimer, Ajay Vallakati, Elyse Redder, Courtney M. Campbell, Nidhi Sharma, Yvonne Efebera, Amro Stino

**Affiliations:** ^1^Division of Hematology-Oncology, Department of Internal Medicine, The Ohio State University Wexner Medical Center, Columbus, OH, United States; ^2^Division of Cardiology, Department of Internal Medicine, The Ohio State University Wexner Medical Center, Columbus, OH, United States; ^3^Division of Nephrology, Department of Internal Medicine, The Ohio State University Wexner Medical Center, Columbus, OH, United States; ^4^Division of Neuromuscular Medicine, Department of Neurology, The Ohio State University Wexner Medical Center, Columbus, OH, United States; ^5^Oncology Rehabilitation, The Ohio State University James Cancer Center, Columbus, OH, United States; ^6^Cardiovascular Division, Cardio-Oncology Center of Excellence, Washington University in St. Louis, St. Louis, MO, United States; ^7^Ohio Health, Department of Hematology/Oncology and Blood and Marrow Transplant, Columbus, OH, United States; ^8^Division of Neuromuscular Medicine, Department of Neurology, The University of Michigan Medical School, Ann Arbor, MI, United States

**Keywords:** amyloidosis, multidisciplinary clinic, transthyretin amyloid, AL amyloidosis, personalized medicine

## Abstract

Amyloidosis refers to a group of conditions where abnormal protein—or amyloid—deposits in tissues or organs, often leading to organ malfunction. Amyloidosis affects nearly any organ system, but especially the heart, kidneys, liver, peripheral nervous system, and gastrointestinal tract. Neuromuscular deficits comprise some of its ubiquitous manifestations. Amyloidosis can be quite challenging to diagnose given its clinical heterogeneity and multi-system nature. Early diagnosis with accurate genetic and serologic subtyping is key for effective management and prevention of organ decline. In this review, we highlight the value of a multidisciplinary comprehensive amyloidosis clinic. While such a model exists at numerous clinical and research centers across the globe, the lack of more widespread adoption of such a model remains a major hindrance to the timely diagnosis of amyloidosis. Such a multidisciplinary care model allows for the timely and effective diagnosis of amyloidosis, be it acquired amyloid light amyloidosis (AL), hereditary transthyretin amyloidosis (hATTR), or wild type amyloidosis (TTR-wt), especially in the current era of personalized genomic medicine. A multidisciplinary clinic optimizes the delivery of singular or combinatorial drug therapies, depending on amyloid type, fibril deposition location, and disease progression. Such an arrangement also helps advance research in the field. We present our experience at The Ohio State University, as one example out of many, to highlight the centrality of a multi-disciplinary clinic in amyloidosis care.

## Introduction

Amyloidosis refers to a constellation of disorders characterized by abnormal protein deposition (1, 2). Conformational changes in protein structure result in protein aggregation and fibril formation (3, 4). These fibrils infiltrate end organs, leading to organ failure and even death. Depending on the organ or tissue affected, the onset of symptoms may be sudden and noticeable, or subtle and insidious. Nearly 4,000 people are diagnosed with amyloidosis each year in the United States. There are different subtypes of amyloidosis (5), with the most common being immunoglobulin-derived acquired light chain amyloidosis (AL amyloidosis) and transthyretin (TTR) amyloidosis, the latter being the most common hereditary form. Amyloidosis presents with a myriad of clinical complaints.

While clinical findings in amyloidosis are by no means unique, when presented together, they should raise concern for the condition and prompt a more aggressive disease screen. Potentially concerning findings including nephrotic syndrome in the absence of hypertension or diabetes; heart failure with preserved ejection fraction (in the absence of hypertension); unexplained hepatomegaly; macroglossia; periorbital purpura (seen in 15% of patients); recurrent unilateral or bilateral carpal tunnel syndrome; unexplained autonomic neuropathy, rapidly aggressive sensorimotor polyneuropathy; and neuromyopathy (6). Other findings seen in amyloidosis include acral swelling, splenomegaly, and vitreous opacification, as well as spinal stenosis. Non-neurologic manifestations like cardiomyopathy or nephropathy are often the most disabling deficits.

Neurologists are sometimes the first providers to encounter amyloidosis due to the frequent and often early involvement of peripheral somatic and autonomic nerves. Amyloid peripheral neuropathy is typically rapid in onset (progressing to upper extremities within 4–5 years) and can cause proximal upper and lower limb weakness. Through this review, we aim to focus on the neuromuscular manifestations of amyloidosis, specifically to allow for a timelier diagnosis for patients who first present with neurologic manifestations. We also highlight the value of a multidisciplinary amyloidosis clinic in our current era of personalized medicine. We also provide a brief overview of the pathophysiologic, diagnostic, and therapeutic nuances of AL-, TTR-, wild type-, and β-2-microglobulin- associated (β2M) amyloidosis.

## Overview of types of amyloidosis

### Acquired light chain amyloidosis

Acquired light chain (AL) amyloidosis is a rare plasma cell dyscrasia that affects 10 patients per million person-years (7). In AL amyloidosis, a B-cell clone produces a toxic light chain that forms insoluble amyloid fibrils (8). These insoluble amyloid fibrils then deposit in different end organs that lead to multiorgan failure. AL amyloidosis commonly affects the kidneys, resulting in nephrotic syndrome. Amyloid cardiomyopathy is the second most common presenting manifestation. Other organ systems that may be involved include the peripheral and autonomic nervous systems, the vasculature, the liver and gastrointestinal tract, and soft tissues. In systemic AL amyloidosis, the plasma cell clone is usually small (median infiltrate typically 10%). Common cytogenetic abnormalities are t(11;14) and gain 1(q21), seen in 50% of patients. Cardiac involvement is the major predictor of survival, and is used to stratify subjects in clinical trial design (9, 10). Cardiac MRI is usually recommended to evaluate for cardiac involvement, but cannot distinguish AL from TTR forms of amyloidosis, and so an endomyocardial biopsy may be required. Nerve conduction testing and electromyography should be pursued to evaluate for a neuropathy and carpal tunnel syndrome. Furthermore, the presence of a neuromyopathy pattern on testing should raise added concern for AL Amyloidosis. A tissue biopsy is often necessary to establish the diagnosis of AL amyloidosis, preferably in the form of bone marrow or fat pad aspirate, although nerve biopsy is increasingly used. If possible, biopsy of the affected organ is preferred, including, for example, the endomyocardium or kidney itself. AL amyloidosis cases should be referral to a specialized amyloidosis center, like a comprehensive amyloid clinic, for additional workup and treatment.

While there have been advances in the treatment of AL amyloidosis, major unmet therapeutic needs still remain (11). The current therapeutic approach for AL amyloidosis (AL) rests on the targeting of toxic amyloidogenic light chain-producing plasma cells followed by autologous stem cell transplantation in select patients. The goal of therapy is to achieve a targeted reduction in the affected free light chains to prevent further organ damage (3, 12). The introduction of the anti-CD 38 antibody daratumumab represents a major advance in treatment, and has led to sustained therapeutic response in many patients (13, 14). The phase 3 Andromeda trial (15–17) randomized subjects to receive either six cycles of bortezomib, cyclophosphamide, and dexamethasone (VCD) either alone or in combination with subcutaneous daratumumab followed by single-agent daratumumab every 4 weeks for up to 24 cycles. At last follow up, hematologic complete remission rates were higher with the combinatorial daratumumab+VCD regimen than with VCD alone (59 vs. 19%). Cardiac response rates were higher with daratumumab+VCD than with VCD alone at 6 mo (42 vs. 22%) and 12 mo (57 vs. 28%), while renal response rates were 54% vs. 27% at 6 mo and 57% vs. 27% at 12 mo, respectively. This trial helped usher in FDA approval of the quadruplet regimen (d-VCD) in 2011, which is now the standard of care. Newer ongoing trials include anti-amyloid fibril antibody CAEL-101 (18) as well as Birtamumab (19).

### Hereditary transthyretin amyloidosis (hATTR)

Hereditary transthyretin (hATTR) amyloidosis is an autosomal dominant genetic disorder characterized by the abnormal and excessive expression of TTR protein (produced mainly in the liver), which mis-folds and eventually aggregates into fibrils that deposit in numerous end organs. The disorder often manifests with peripheral somatic and autonomic neuropathy as well as restrictive cardiomyopathy. The disease usually progresses over a five to ten year period from time of diagnosis to demise if untreated, often due to congestive heart failure (20). TTR amyloidosis is the most prevalent and well-characterized of the three major forms of familial amyloid polyneuropathy, the others being apolipoprotein A1-associated and gelsolin-associated amyloid neuropathy. In the normal state, TTR functions as a transport protein of thyroid hormone and retinol (vitamin A), hence, the name, transthyretin. Over 120 pathogenic TTR mutations (21) are now described, with the most common worldwide variant (*Val30Met*) carrying a frequency ranging from 1:538 in Portugal and 1:100,000 to 1:1,000,000 in the United States. hATTR has early and late onset forms, with early onset hATTR having more autonomic involvement (21). The *Val142Ile* mutation is the most prevalent form in the US and is primarily cardiac, with 3% of African Americans in the US carrying the mutation (22, 23). Interestingly, L-homocysteine, an established cardiovascular risk factor, has been shown in *in vitro* studies to impair cardiomyocyte function and worsen tetramer stability in L55P variant TTR models, which are associated with early onset cardiomyopathy (24). In addition to affecting the peripheral nervous system and heart, hATTR also affects the renal, GI, hepatic, and ophthalmic systems. Initial evaluation rests largely on clinical suspicion, especially in the setting of rapidly progressive peripheral neuropathy, unexplained cardiomyopathy (especially restrictive), recurrent carpal tunnel syndrome despite surgery, and/or autonomic dysfunction. Helpful tests include nerve conduction studies as well as cardiac imaging, which includes echocardiography, cardiac MRI, and Tc-99-m pyrophosphate (PYP) scintigraphy. PYP scanning has at times obviated the need for endocardial or other tissue biopsy given its high sensitivity (97%) and specificity (100%) in diagnosing hATTR (25–27).

Genetic testing for hATTR should be pursued in the setting of a suggestive family history. This can be performed using commercial full-gene sequencing and deletion/duplication analysis *via* next generation sequencing (NGS) technology. Such technology focuses on clinically relevant regions in each gene and assesses both coding exons as well as adjacent intronic regions. Such assays carry high sensitivity and specificity, but variants of undetermined significance must be interpreted in the context of a given clinical phenotype to ensure accuracy. The clinical presentation of hATTR depends on the pathogenic mutation and can manifest with cardiac—or neuropathic- predominant disease.

**Figure 1:**
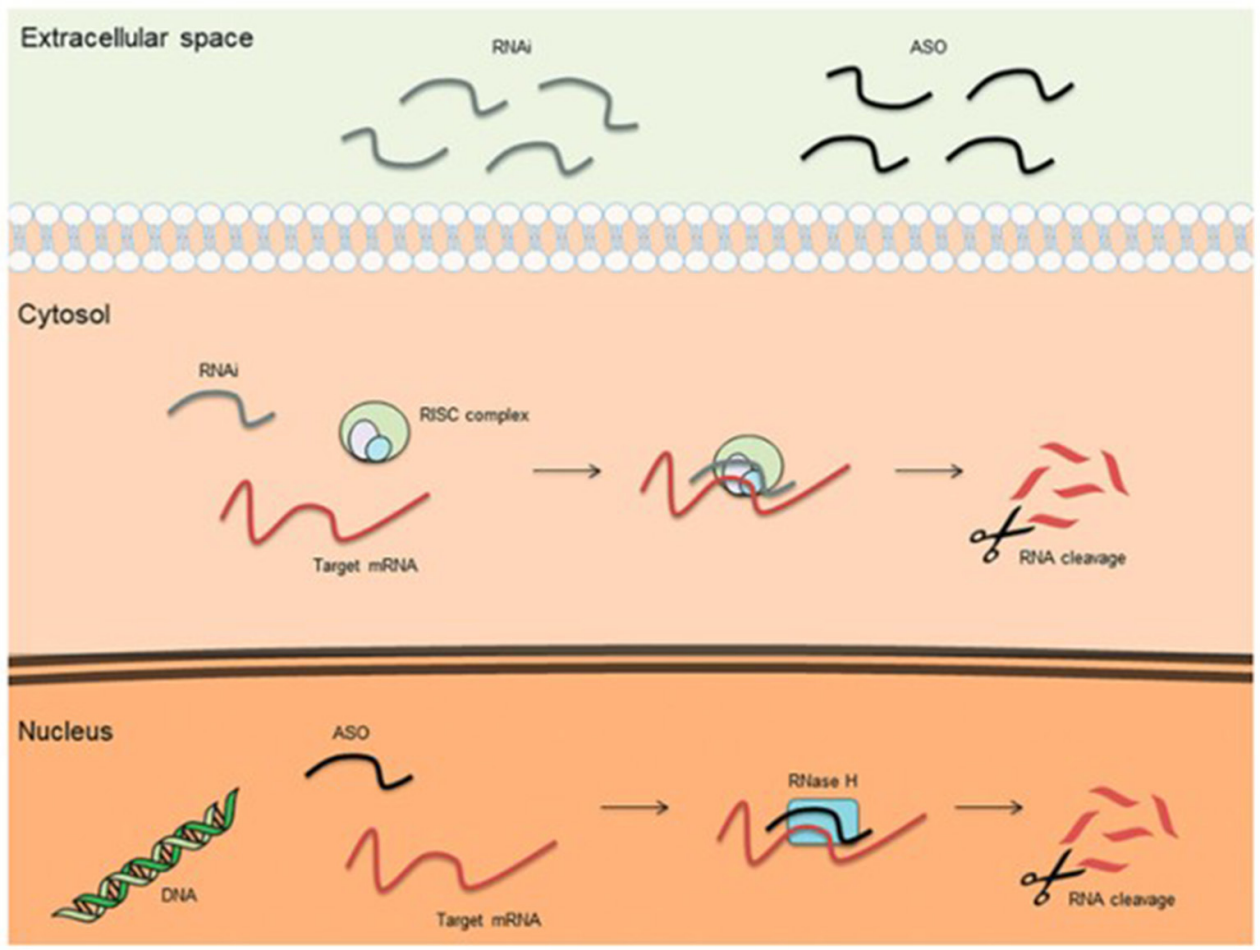
Basic mechanisms of action for therapeutic antisense oligonucleotides (ASOs) and RNA interference (RNAi) (38).

Mechanisms of treatment of hATTR amyloidosis involve native tetramer stabilization, RNA interference, and antisense oligonucleotide therapy. The stabilization of amyloid tetramers is one mechanism of reducing amyloid fibril formation and end-organ disease burden. Tafamidis gained FDA approval for the treatment of hATTR-associated cardiomyopathy, having shown improved survival and reduced hospitalizations, as compared to placebo (28). Diflunisal, an oral nonsteroidal anti-inflammatory drug, also carries therapeutic promise both with regards to arresting neuropathic and cardiac disease progression, but does not carry FDA approval for either indication (29, 30). New to the treatment landscape is the arrival of RNA interference (RNAi) and antisense oligonucleotide (ASO) therapies (Figure 1). RNAi therapies consist of oligonucleotides that down-express target proteins by rapidly degrading mRNA in the cytoplasm. Such therapies are ideal for toxic gain-of-function diseases, like hATTR. Small interfering RNAs (siRNAs) are single or double-stranded RNAs (complementary to the mRNA targeted for degradation) that activate an RNA-induced silencing complex (RISC). The RIS complex, in turn, activates degradation of the target mRNA. Patisiran (31) is a small interfering RNA molecule complementary to the 3' untranslated region of both wild-type and mutant mRNA TTR, and degrades both in the cytosol. A phase 3 trial demonstrated that intravenous Patisiran, at 18 months, improved or stabilized multiple neuropathy outcome measures, including both somatic and autonomic function, leading to its FDA approval for the treatment of hATTR-associated polyneuropathy. Most recently, Vutrisiran, a subcutaneously administered RNAi therapy that is given every 3 months, also received FDA approval for the treatment of hATTR-associated polyneuropathy (32). On the other hand, antisense oligonucleotides (ASOs) are short, single-stranded deoxyribonucleic acids that degrade target RNA by binding primarily to target pre-mRNA in the nucleus (as opposed to the cytoplasm). ASO-mediated degradation occurs *via* the enzyme RNase H. The ASO therapy Inotersen (33), like Patisiran, binds to both wild-type and mutant TTR, induces a measurable decrease in mutant TTR level, and also received FDA-approval for the treatment of hATTR-associated polyneuropathy. A phase 3 clinical trial showed that weekly subcutaneous Inotersen arrests neuropathy progression. In addition to tetramer stabilization and gene silencers, interest has also arisen for polyphenols as potential barriers to amyloidogenesis, whether in the form of oleuropein aglycone (34), derived from extra virgin olive oil, or epigallocatechin-gallate, derived from green tea (35, 36). Oleuropein aglycone was also shown in an *in vitro* study to attenuate amyloid associated cytotoxicity in a hereditary β2M systemic amyloidosis model (37).

### Wild type TTR amyloidosis (TTRwt)

Wild type TTR amyloidosis (TTRwt) represents a unique subset of amyloidosis that is acquired (unlike hATTR) and disproportionately affects older men (39). In TTRwt patients, the naturally occurring TTR protein becomes increasingly unstable over time and forms amyloid deposits that accrue mainly in the heart, causing such cardiac manifestations as heart failure with preserved ejection fraction, aortic stenosis, atrial fibrillation, and angina, as well as syncope. Patients may also manifest with carpal tunnel syndrome and/or peripheral neuropathy. The previously discussed amyloidosis workup should be pursued, which includes, but is not limited to, cardiac imaging, as well as tissue biopsy, if/when indicated. Genetic testing for hATTR should also be pursued. Tafamidis is FDA-approved for the treatment of TTRwt-associated cardiomyopathy.

### β-2-microglobulin—associated (β2M) amyloidosis

β2M amyloidosis is a, less appreciated systemic manifestation of amyloidosis that has been recognized for over 80 years (40). It was formerly called dialysis related amyloidosis, due its disproportionate occurrence amongst patients with end stage renal disease (ESRD) on hemodialysis. Dating back to the 1970s, it was noticed that ESRD patients had a high prevalence of carpal tunnel syndrome. In healthy subjects, β2M amyloid fibrils are constantly and naturally produced in the body and renally cleared. In ESRD patients, these fibrils accrue in the body, and deposit in such regions as the spinal column (41) and carpal tunnel as well as the arterioles, venules, heart, and GI tract. Risk factors include increased age, increased hemodialysis duration, and advanced renal injury. Renal transplant and the use of high-flux HD ameliorate fibril deposition.

## Multidisciplinary amyloid care

### The need for a comprehensive amyloid clinic

Multi-disciplinary care is now considered the gold standard in the care of complex diseases, particularly in our current era of personalized medicine. The value and cost-effectiveness of multidisciplinary care spans numerous disorders, ranging from chronic kidney disease (42) to congestive heart failure (43) to amyotrophic lateral sclerosis (44). For example, multidisciplinary care improves mortality in ALS patients (45). Sarcoidosis is another relatively rare multi-organ disease, highly variable in its clinical presentation and challenging in its diagnosis, also best managed in a multidisciplinary setting (46, 47).

Specifically with regards to hematologic-oncologic disorders such as amyloidosis, multidisciplinary care improves patient satisfaction and outcome (48). Historically, amyloidosis has been underrecognized and uncoordinated in its care. It is marred by delay in diagnosis and treatment. Three-quarters of patients require at least 12 months to reach a definitive diagnosis (49). The lack of specialty care means that patients get referred to multiple providers, further adding to cost and diagnostic delay (50). Multi-disciplinary amyloid care is still in its infancy, but is growing, whether for AL amyloidosis (51), TTR amyloidosis (52), or amyloid cardiomyopathy (53).

Accordingly, the comprehensive amyloid clinic at The Ohio State University Wexner Medical Center was created to improve the quality of care and outcome of amyloidosis patients. To reiterate, this is but one of numerous such centers already in existence across the globe and serves as but one example. The hematology team recognized the importance of early diagnosis and treatment, but also sought to create a more collaborative and unified approach, one focused on patient care, education, and research. The purpose of the comprehensive amyloid clinic is to provide patients with efficient, appropriate, and timely care. Different specialties participate in patient evaluation and care with the goal of managing symptoms, reducing amyloid protein deposition, and supporting affected organs. We integrate basic and translational research into our clinical work. There is a paucity of published data on the role of academic multidisciplinary specialty clinics in amyloidosis. Comparisons about race and socioeconomic class only assess basic access to care and disease heterogeneity. Patient survival remains poor (across race) for most patients, as shown in phase 3 trials (54), but we believe that this can be addressed through multi-disciplinary care.

### Program structure

**Figure 2:**
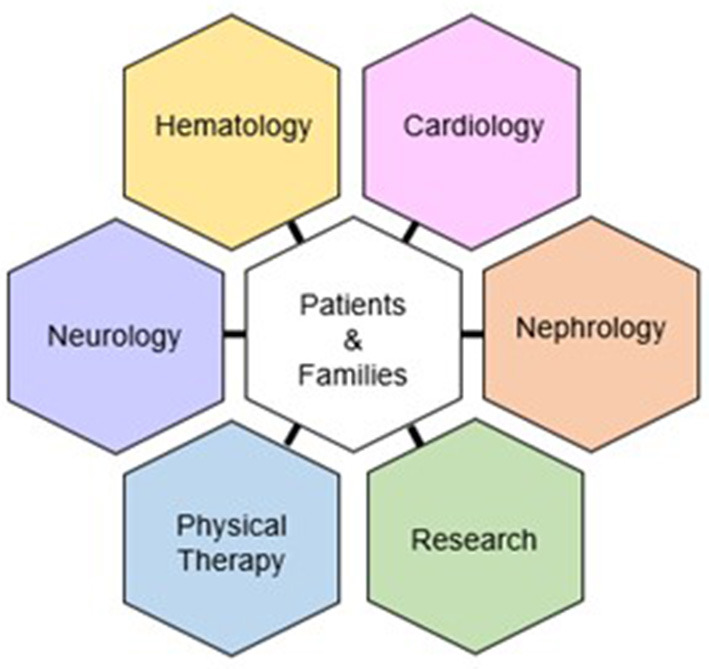
Organizational structure of the Ohio State University Comprehensive Amyloidosis Clinic.

Our program is directed by board-certified hematologists with specialty interest and training in amyloidosis. Alongside our hematology-oncology core is a multi-disciplinary team comprised of nephrologists, neurologists, cardiologists, and physical therapists (Figure 2). The clinic is predicated on (1) an interdisciplinary and collaborative arrangement, where providers from each specialty participate in a post-clinic roundtable to address diagnosis and management. In addition, team members serve as co-investigators in trials involving clinic patients. (2) a one-stop design, where patients arrive at one central location and are seen in designated rooms *via* a rotating schedule of providers; and (3) ancillary staff that educates patients and schedules necessary testing (including insurance prior authorization) and assists with study recruitment and data/specimen collection.

### Patient workflow

**Figure 3:**
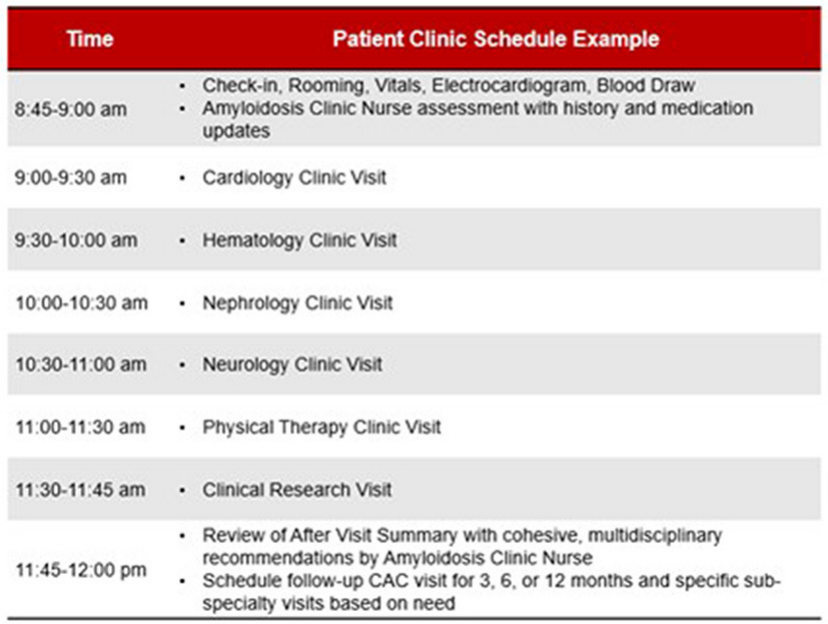
Workflow for the Ohio State University Comprehensive Amyloidosis Clinic.

**Figure 4:**
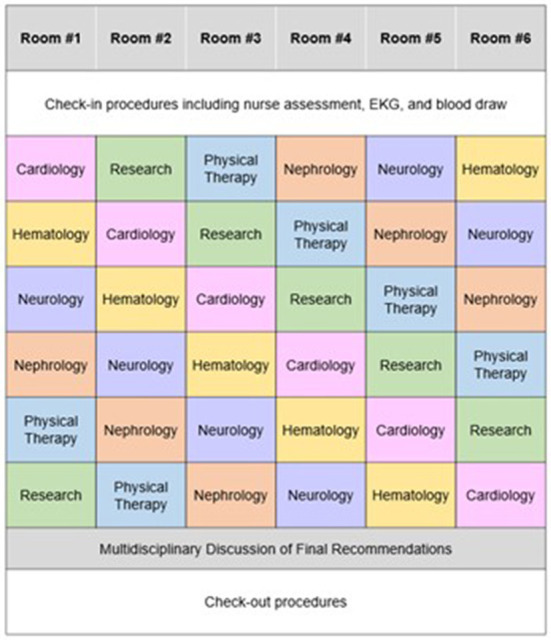
Example schedule for a patient during a visit to the Ohio State University Comprehensive Amyloidosis Clinic.

The Ohio State comprehensive amyloid clinic was the first of its kind in Ohio, and one of the first in the Midwest to provide multidisciplinary amyloidosis care in a single location. The clinic runs every 2 weeks. Up to 7 patients are scheduled per clinic day. Patients all arrive at the start of the clinic day (same time) and are given separate fixed room assignments. Each patient is scheduled to see 5 separate providers in his/her room— hematology, cardiology, neurology, nephrology, and physical therapy (each visit is assigned 40 min), for a total visit duration of 3 h per patient (Figure 3). The workflow is designed to streamline delivery of care and facilitate management for patients and caregivers. Upon patient arrival, an assigned primary registered nurse and two personal care assistants check patients in and then obtain vitals, baseline electrocardiogram, and baseline labs, prior to assigning patient rooms on the master clinic schedule. RNs specify if patients have specific check-in requirements. Providers rotate through each clinic based on availability, with the nurse and patient care assistants responsible for the master schedule (Figure 4). Finally, a sit-down multi-disciplinary discussion is held after clinic to discuss final recommendations.

**Figure 5:**
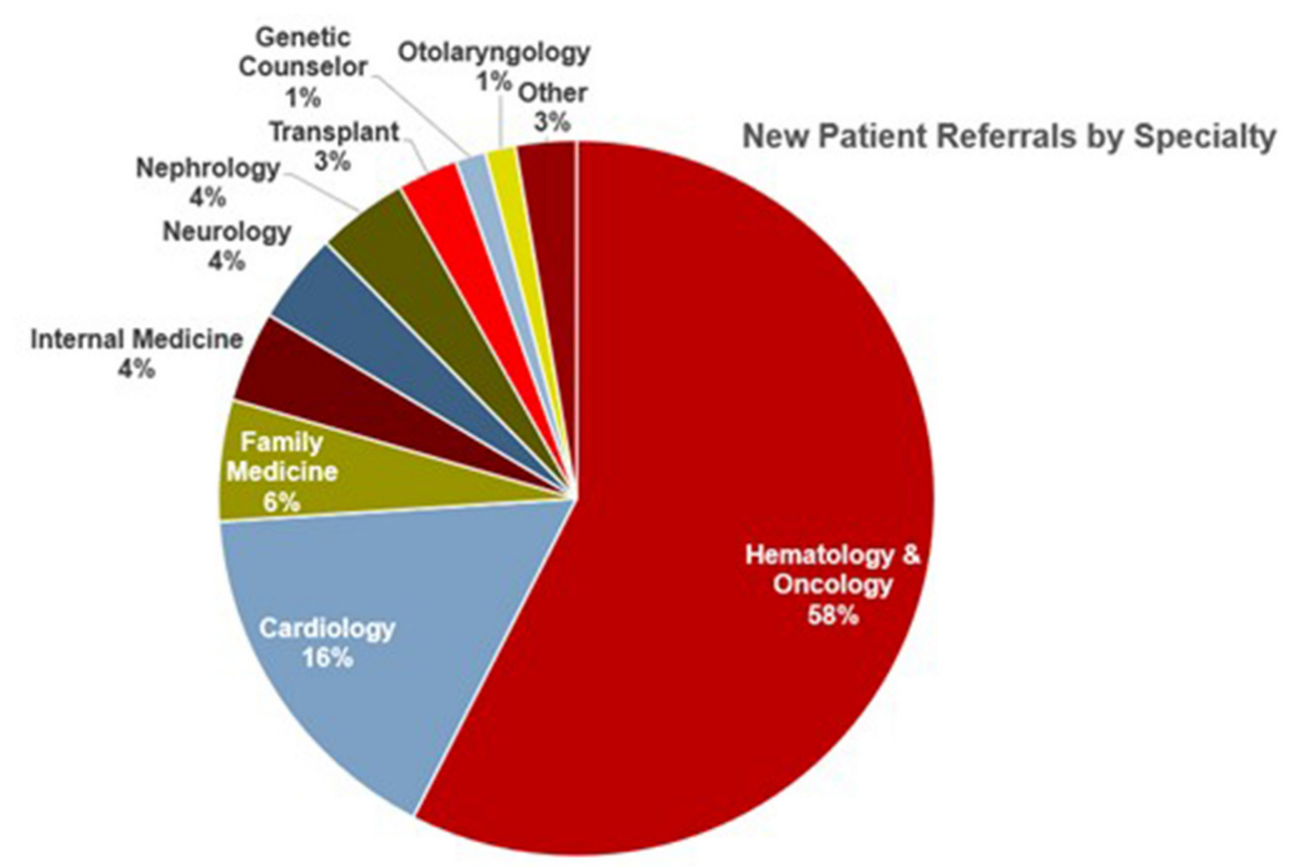
Source of referrals to the Ohio State University Comprehensive Amyloidosis Clinic, arranged by specialty.

Since its inception, the comprehensive amyloid clinic has seen more than 200 patients. Over time, we have significantly reduced new referral wait times. Collaboration between different specialties has been fruitful, with two out of every five referrals now coming from specialties other than hematology-oncology (Figure 5). Furthermore, the clinic has gained local and national recognition with an increase in referrals, with nearly one-fifth of patients coming from outside centers.

## Representative cases

### Case 1

Patient 1 is a male in his 50s referred to cardiology and neurology for worsening dyspnea, acral edema, and neuropathy, respectively. He had undergone a cardiac MRI, which showed diffuse myocardial enhancement with myocardial nulling, an ejection fraction of 47%, and an interventricular septal thickness of 2.3 cm. He was then referred to the comprehensive amyloid clinic. He underwent an extensive workup for AL amyloid and plasma cell markers, which were negative. Technetium pyrophosphate scan (PYP-99) revealed diffuse pyrophosphate uptake in the left ventricular myocardium, consistent with a diagnosis of hATTR cardiomyopathy. Given his age and potential eligibility for heart transplant, an endomyocardial biopsy was done, which showed amyloid deposits in the interstitium. The sample was sent for mass spectrometry, which confirmed a peptide profile consistent with ATTR (transthyretin)-type amyloid deposition. A genetic panel was sent for mutational analysis, and he was found to have a TTR (transthyretin) Val 142Ile mutation. Within 2 months of presenting to our institution, he had a definitive diagnosis. Treatment was initiated with fibril stabilizers diflunisal, doxycycline and actigall. Since he had neuropathic symptoms, RNAi therapy with Patisiran was initiated, which improved his symptoms. Due to cardiac involvement, Tafamidis was also subsequently added. With these interventions, he had significant improvement in his cardiac status and was referred for a heart transplant.

### Case 2

Patient 2 is a male in his late 70s who presented with shortness of breath after undergoing a spinal decompression and fusion. A transthoracic echocardiogram showed a depressed ejection fraction of 35–40% and an increased interventricular septal thickness of 1.6 cm. As part of a cardiac ischemic evaluation, a pharmacological stress test was performed, which was normal. He was referred to our comprehensive amyloid clinic. A cardiac MRI was performed, which showed nearly diffuse late gadolinium enhancement and left atrium, consistent with amyloid cardiomyopathy. He then underwent a fat pad biopsy that showed amyloid deposit. Mass spectrometry revealed a peptide profile consistent with AL (lambda)-type amyloid deposition. Bone marrow biopsy revealed a hypercellular marrow with 20% lambda restricted plasma cells. Bloodwork showed elevated lambda light chain markers as well as elevated troponin and NT-proBNP. He was deemed stage III disease per the Mayo criteria. Within 2 weeks of initial presentation, he was formally diagnosed with AL amyloidosis and initiated on Cyclophosphamide, Bortezomib and Dexamethasone (CyBorD) therapy. He achieved a hematological partial remission after 6 cycles of CyBorD. He was not a stem cell candidate due to advanced cardiac involvement and advanced age. He was placed as part of a daratumumab maintenance on clinical trial.

### Role of research

Concurrent with clinical operations, the comprehensive amyloid clinic has focused on four key research questions, namely quality of life, environmental exposures and risk factors, treatment modalities, and overall survival. In addition, the comprehensive amyloid clinic has been involved in numerous international multi-center natural history and drug trials. Given the clinical heterogeneity of amyloidosis and the ubiquity of its clinical symptoms, more needs to be known regarding its pathology to allow for more precise understanding of disease pathogenesis and the development of targeted therapeutics.

## Conclusions

Amyloidosis is a multi-system disease that is both challenging in its diagnosis and rapid in its progression. Powerful diagnostic tools and therapeutic options now allow for improved patient outcome and quality of life. Through a multidisciplinary care model, synchronized clinical care can be delivered in a more timely and personalized fashion. We believe that such a clinic arrangement improves access and outcome for amyloidosis patients and is integral to the delivery of personalized medicine.

## Author contributions

NB contributed to topic formulation, data collection, manuscript writing, and manuscript revision. RK, SP, MI, MF, AV, ER, CC, and NS contributed to manuscript writing. YE contributed to topic formulation and manuscript writing. AS contributed to topic formulation, manuscript writing, and manuscript revision. All authors contributed to the article and approved the submitted version.

## Funding

CC was supported, in part, by the National Center for Advancing Translational Sciences of the National Institutes of Health under Grant Numbers TL1TR002735 and UL1TR001450. AS was supported by the GBS-CIDP Foundation.

## Conflict of interest

The authors declare that the research was conducted in the absence of any commercial or financial relationships that could be construed as a potential conflict of interest.

## Publisher's note

All claims expressed in this article are solely those of the authors and do not necessarily represent those of their affiliated organizations, or those of the publisher, the editors and the reviewers. Any product that may be evaluated in this article, or claim that may be made by its manufacturer, is not guaranteed or endorsed by the publisher.
